# Hyperreflective foci and subretinal drusenoid deposits as predictors of exudation in intermediate AMD eyes with contralateral neovascular AMD

**DOI:** 10.1038/s41433-026-04617-8

**Published:** 2026-07-18

**Authors:** Enrico Bernardi, Usha Chakravarthy, Katherine A. Muldrew, Ruth E. Hogg, David M. Wright, Martin Zinkernagel, Tunde Peto, Lajos Csincsik

**Affiliations:** 1 Department of Ophthalmology, Belfast Health and Social Care Trust, Belfast, UK; 2 Department of Ophthalmology, Inselspital, Bern University Hospital, University of Bern, Freiburgstrasse 15, CH-3010 Bern, Switzerland; 3 Centre for Public Health, School of Medicine Dentistry and Biomedical Sciences, Queen’s University Belfast, Belfast, UK

**Keywords:** Prognostic markers, Predictive markers, Retinal diseases

## Abstract

**Objective:**

To examine the prognostic significance of intraretinal hyperreflective foci (HRF) and subretinal drusenoid deposits (SDD) as predictors of atrophy presence and progression to exudative macular neovascularisation (eMNV) in the unaffected second eye of participants with eMNV in the first eye.

**Methods:**

Post hoc prognostic analysis on iAMD eyes from the prospective EDNA cohort. SD-OCT scans of 459 study eyes were graded for presence of HRF, SDD, and their characteristics, including location within retina (inner, outer, or both), proximity to irregular elevation of RPE (adjacent, remote or both), and SDD load (mild or moderate to severe). The cohort was subdivided in four follow up groups, iAMD without focal regions of atrophy (FoA), persistent or new onset FoA, eMNV alone and eMNV with FoA.

**Results:**

Of the 459 included eyes, 234 (51.0%) had HRF and 273 (60.0%) had SDD at baseline. Persistent or new onset FoA was seen in 83, 66 progressed to eMNV alone and 38 to eMNV with FoA, and 272 had no worsening of iAMD status. Presence of HRF and especially a higher SDD load were associated with a significantly faster progression to exudation. A higher SDD load doubled the hazard of progression to any eMNV compared to eyes with a mild load or without SDD. This hazard was highest in eyes with the presence of both HRF and SDD. HRF were associated with a lower BCVA.

**Conclusions:**

HRF and SDD are consistently observed in high risk AMD eyes and characterising their distribution and load might allow differentiation of their contributions to risk of progression to late stage features of atrophy and exudation.

## Introduction

Age-related macular degeneration (AMD) is a multifactorial degenerative disease resulting in a gradual loss of central vision [1]. It is the leading cause of blindness in the industrialised world and the fourth worldwide [2, 3]. AMD progresses through early, intermediate, and advanced stages, with the advanced forms being neovascular AMD (nAMD), which can be non-exudative or exudative (macular neovascularisation, eMNV), and geographic atrophy (GA), characterised by the degeneration of retinal pigment epithelium (RPE) and photoreceptors [4]. Early detection and monitoring of AMD progression are essential to prevent or delay vision loss arising from eMNV which can be acute and rapid, and thus optical coherence tomography (OCT) has become a crucial tool in the diagnosis of this process [5, 6]. Both eMNV and GA can occur in the same eye, in the same individual, with GA in one eye and eMNV in the other, indicating that they share common pathogenetic pathways. In a large electronic medical record study it was observed that GA in the first eye increased the probability of GA developing in the fellow eye by 11.2% and eMNV by 8.5% [7]. Conversely, the presence of eMNV in one eye resulted in a higher percentage that developed exudation in the fellow eye (15.2%) compared to those that developed GA (4.1%) [7]. However the factors that mediate divergence of these pathways remain largely unknown.

Intraretinal hyperreflective foci (HRF) are among the OCT biomarkers that have attracted attention because of their link to AMD progression. They are well-circumscribed lesions within the neurosensory retina with a reflectivity similar to the retinal pigment epithelium (RPE) and often correspond to hyperpigmentation on colour fundus photography [8, 9]. Studies have linked the presence of HRF to an increased risk of progression to both nAMD and GA [10–12]. Although HRF have been identified as significant indicators of AMD progression, studies show considerable variability in the strength of their associations with disease outcomes. This heterogeneity may be attributed to differences in HRF characteristics, such as their location within the retina and their spatial relationship to other retinal structures [13]. For instance, HRF in the outer retinal layers have been more commonly associated with progression to GA [14].

Similarly, subretinal drusenoid deposits (SDD), also known as reticular pseudodrusen, are a novel biomarker which has been increasingly studied in relation to AMD. First described over 30 years ago, they are a subretinal deposition of drusenoid material often arranged in a reticular pattern and characteristics similar to soft drusen, which are ordinarily found on the inner portion of Bruch’s membrane [15–17]. Notably, they have been increasingly associated with GA [12, 18].

Understanding how the presence of HRF and SDD could impact the development of new atrophy or progression to eMNV is necessary for improving prognostic tools and enhancing treatment strategies. An analysis was therefore conducted on a repository of data from the early detection of neovascular AMD (EDNA) study which was aimed at examining the prognostic significance of HRF and SDD after detailed characterisation of these two biomarkers on spectral domain optical coherence tomography (SD-OCT).

## Materials and methods

Secondary grading and post hoc observational prognostic modelling of the SD-OCT repository of the EDNA study (ISRCTN: 48855678). EDNA was a multicentre, prospective, cohort, comparative diagnostic accuracy study conducted in 24 ophthalmology departments within UK NHS hospitals that enroled over 552 participants with newly diagnosed unilateral eMNV, with informed consent obtained from all subjects. The high-risk fellow eye of EDNA participants (study eye) was observed until progression to eMNV or up to 3 years. The aim of EDNA was to identify the most appropriate monitoring strategy to detect eMNV when it occurred in the unaffected study eye using pragmatic tests of function and imaging. Onset of eMNV in the study had therefore been determined at or close to the time of progression to exudation through intensive monitoring and recorded with high fidelity in the database. The instruments and protocols used for OCT capture of macular images are shown in Supplementary Table 1S, and their heterogeneity was considered when performing the analysis.

### OCT grading

Imaging systems and scan parameters were reported in previous publications involving the EDNA database [12, 19]. For the present analysis, a further detailed grading was performed in which SD-OCT scans were assessed for the presence of HRF and SDD.

HRF were graded as present when scattered, punctiform, focal hyperreflective lesions equal to or greater than the reflectivity of the RPE were observed within the neurosensory retina. The anatomical location of HRF within the retina was also documented as outer retinal, inner retinal, or both and proximity to areas of IE was recorded as adjacent, remote, or both.

SDD were defined as accumulations of moderate to highly hyperreflective material forming either sharp peaks or broad, rounded elevations located internal to the RPE. Further characteristics of SDD, including load, location with respect to the fovea and proximity to areas of irregular elevation of retinal pigment epithelium (IE) were recorded. SDD load was based on the proportion of B scans of the OCT volume scans in which SDD were detected; these were classified as mild ( ≤ 25% of B scans), moderate (between 26% and 75% of B scans) and severe ( ≥ 76% of B scans). Location was defined as sub-foveal if confined to the central subfield of the ETDRS grid, or extra-foveal if the central subfield was not involved. Proximity to areas of IE was defined as adjacent, remote or both.

Images were evaluated either at the final study visit for eyes that did not progress to eMNV, or at the visit when eMNV was first identified. Each OCT volume scan was assessed for the presence of OCT signal hypertransmission into the choroid (HyperTC) through the RPE/Bruch’s membrane complex, and used as a marker for focal atrophy (FoA). Consistent with our previous report, FoA at baseline was defined as a band of hypertransmission measuring ≥40 µm in diameter. We considered the presence of FoA or its development during follow up as a more severe level of iAMD [19]. We therefore divided the study eyes into 4 follow up groups: iAMD without FoA or eMNV, persistent or new onset FoA, progressed to eMNV only, or eMNV with FoA. As the primary outcome in the EDNA study was conversion of the iAMD eye to eMNV (detected in 104 of 459 in the present sample), this data was available for time to progression analyses as it was recorded in the EDNA dataset. Best corrected visual acuity (BCVA) was also obtained from the EDNA database for the baseline and the final visit.

### Data management and analysis

Baseline cohort characteristics were summarised across the four outcome categories. Continuous variables were presented as mean (standard deviation [SD]), median (interquartile range [IQR]), and minimum–maximum range. Categorical variables were presented as frequencies and percentages. Between-group differences were assessed using the Kruskal–Wallis rank-sum test for continuous variables and Pearson’s chi-squared test (or Fisher’s exact test where expected cell counts were fewer than five) for categorical variables. A two-sided p-value of <0.05 was considered statistically significant throughout. Due to an uneven distribution among the SDD load groups (only few eyes, *N* = 33, fell into the severe category), the latter two were amalgamated in a “moderate to severe” category ( ≥ 26% of area covered) to streamline the analysis.

The primary outcome was time to development of eMNV. Progression-free survival was estimated using the Kaplan–Meier method for the variables of HRF, SDD load, and their combination as mutually exclusive categories. Survival times corresponding to 50% and 90% event-free probability were derived from the Kaplan–Meier curves and reported with 95% confidence intervals, adjusted for age and sex. The cumulative incidence of eMNV stratified by HRF, SDD and by the combined variable was also assessed.

The association between baseline OCT biomarkers and progression to eMNV was quantified using a multivariable Cox proportional hazards regression. Two separate models were fitted: a model including HRF (present/absent) and SDD load (absent, mild, moderate to severe) as categorical predictors, adjusted for age, sex, choroidal thickness, and scan distance; and a further model using mutually exclusive combined categories of HRF, SDD, and both. To evaluate additional risk factors that may act as confounders, choroidal thickness was added to both models; as only 5 eyes were graded as having a thick choroid, these were excluded from the model since their inclusion rendered the model unstable. An interaction term between HRF and SDD was tested; no statistically significant interaction was identified. Results are reported as hazard ratios (HR) with 95% confidence intervals and corresponding *p*-values. Wald p-values were used for binary predictors and likelihood ratio test *p*-values for ordinal predictors with more than two levels.

The proportional hazards (PH) assumption was evaluated for all covariates using scaled Schoenfeld residuals and the global Cox.zph test (Table 2S). The global test was nominally significant (χ² = 13.83, df = 6, *p* = 0.032), driven primarily by sex (*p* = 0.004). To formally assess whether any hazard ratio varied over time, time-varying coefficient models incorporating a log(t) interaction term were fitted for HRF, SDD load, and sex (Table 3S). None of the interaction terms reached statistical significance (HRF × log[t]: *p* = 0.061; SDD load mild × log[t]: *p* = 0.849; SDD load moderate × log[t]: *p* = 0.077; sex × log[t]: *p* = 0.746), and the magnitude of the sex interaction was negligible (HR = 1.025 per log-time unit, 95% CI 0.88–1.19). The standard Cox model was therefore retained for all primary analyses.

The effect of baseline HRF and SDD on best-corrected visual acuity (BCVA, ETDRS letters) at the final visit was estimated using multivariable linear regression. Model coefficients are presented with 95% confidence intervals.

Despite no systematic exclusion of participants from this analysis, baseline demographic and clinical characteristics were compared to evaluate potential selection bias between participants included in the final analytic cohort (*n* = 459) and those excluded due to missing data or insufficient follow-up (*n* = 93), using the same statistical tests described above. Included and excluded participants differed significantly only with respect to age (mean 77 vs. 79 years; *p* = 0.002), with no significant differences observed for sex, smoking status, hypertension, cardiovascular disease, diabetes, supplement use, or family history (all *p* > 0.05).

All models were adjusted for age and sex. Data analysis and visualisation were conducted using R version 4.3.2 (2023-10-31 ucrt).

## Results

The EDNA database contained 552 unique entries, from which 459 participants were included after discarding those with missing or ungradable baseline or final-visit images. The mean age was 76 ± 8 years, and 57.0% were female (*n* = 265).

Within the cohort, 83 study eyes demonstrated persistent or new onset FoA, 66 progressed to eMNV alone and 38 to eMNV with FoA, with 272 experiencing no worsening of iAMD status. Of the 104 eyes that developed exudative nAMD during time on study, 56 (53.9%) demonstrated a type 1 CNV, 28 (26.9%) a type 2 CNV, and 20 (19.2%) a type 3 CNV (or RAP). The mean and range of BCVA by these groups at baseline and at the last visit is shown in Table 4S in the supplementary materials.

### HRF and progression categories

HRF were observed at baseline in 234 of 459 eyes, and among participants with this feature females were more prevalent (*N* = 146, 62.4%, *p* = 0.039, Pearson’s Chi-squared test). Patients with HRF were significantly older than those without (77 years, SD 7 vs. 75, SD 8, *p* = 0.003, Kruskal-Wallis rank sum test).

Progression to any eMNV (alone or with FoA) by the presence or absence of HRF at baseline and by their proximity to IE and location within retinal layers (outer or inner) is shown in Table 5S. The frequencies of HRF at baseline and distribution within the retina by outcome categories is shown in Table 1. Location both proximal and remote to IE of RPE was more frequent in eyes with any FoA (56.0% in eMNV with FoA and 47.5% in persistent or new onset FoA) compared to iAMD eyes without FoA or eMNV only.

**Table 1:** Frequencies of HRF and distribution within retina by outcome categories.

Outcome categories	*N*	iAMD without FoA *N* = 272^1^	persistent or new onset FoA *N* = 83^1^	eMNV N = 66^1^	eMNV_FoA *N* = 38^1^
**HRF**	459				
NO		178 (65.4%)	9 (10.8%)	33 (50.0%)	5 (13.2%)
YES		94 (34.6%)	74 (89.2%)	33 (50.0%)	33 (86.8%)
**HRF_IE_relation**	171				
Adjacent		25 (43.9%)	26 (42.6%)	8 (28.6%)	8 (32.0%)
Remote		18 (31.6%)	6 (9.8%)	10 (35.7%)	3 (12.0%)
Both		14 (24.6%)	29 (47.5%)	10 (35.7%)	14 (56.0%)
**HRF_location**	234				
Inner retina		3 (3.2%)	0 (0.0%)	2 (6.1%)	0 (0.0%)
Outer retina		70 (74.5%)	43 (58.1%)	24 (72.7%)	20 (60.6%)
Both		21 (22.3%)	31 (41.9%)	7 (21.2%)	13 (39.4%)

Distribution of HRF presence and spatial features across the four outcome categories

**Fig. 1:**
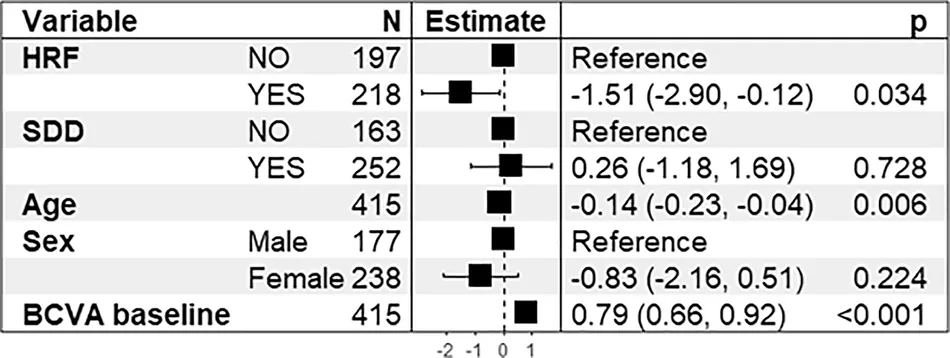
Effect of baseline HRF and SDD on BCVA at end of follow-up. Multivariable linear regression analysis with BCVA at end of follow-up as the outcome variable, adjusted for baseline BCVA. Eyes with baseline HRF had marginally lower BCVA at the end of follow-up compared with those without HRF (β = −1.51 letters, 95% CI −2.90 to −0.12; *p* = 0.034), whereas baseline SDD was not significantly associated with BCVA.

*iAMD* intermediate age-related macular degeneration, *FoA* focal atrophy, *eMNV* exudative macular neovascularisation, *HRF* hyper reflective foci, *IE* irregular elevation; ^1^n (%);

Multivariate linear regression analysis with BCVA as the outcome variable after adjustment for baseline BCVA showed that eyes with HRF at baseline had marginally lower BCVA at end of follow up, with a difference of 1.51 letters (-2.90, -0.12, *p* = 0.034) compared to those without HRF (Fig. 1).

### SDD and progression categories

In the 273 eyes that demonstrated SDD (60.0% of total), females constituted a higher proportion (*N* = 167, 61.2%, *p* = 0.058, Pearson’s Chi-squared test). Comparing participants with SDD to those without the mean age was 78 (SD 7) vs. 74 (SD 8) at baseline and was statistically significant (*p* = 0.001, Kruskal-Wallis rank sum test).

Table 6S shows progression to any eMNV by SDD presence or absence, load, proximity to IE and distribution within the retina at baseline, and Table 7S shows frequencies of SDD, load and distribution by the 4 outcome categories.

### Survival and hazard analysis

**Fig. 2:**
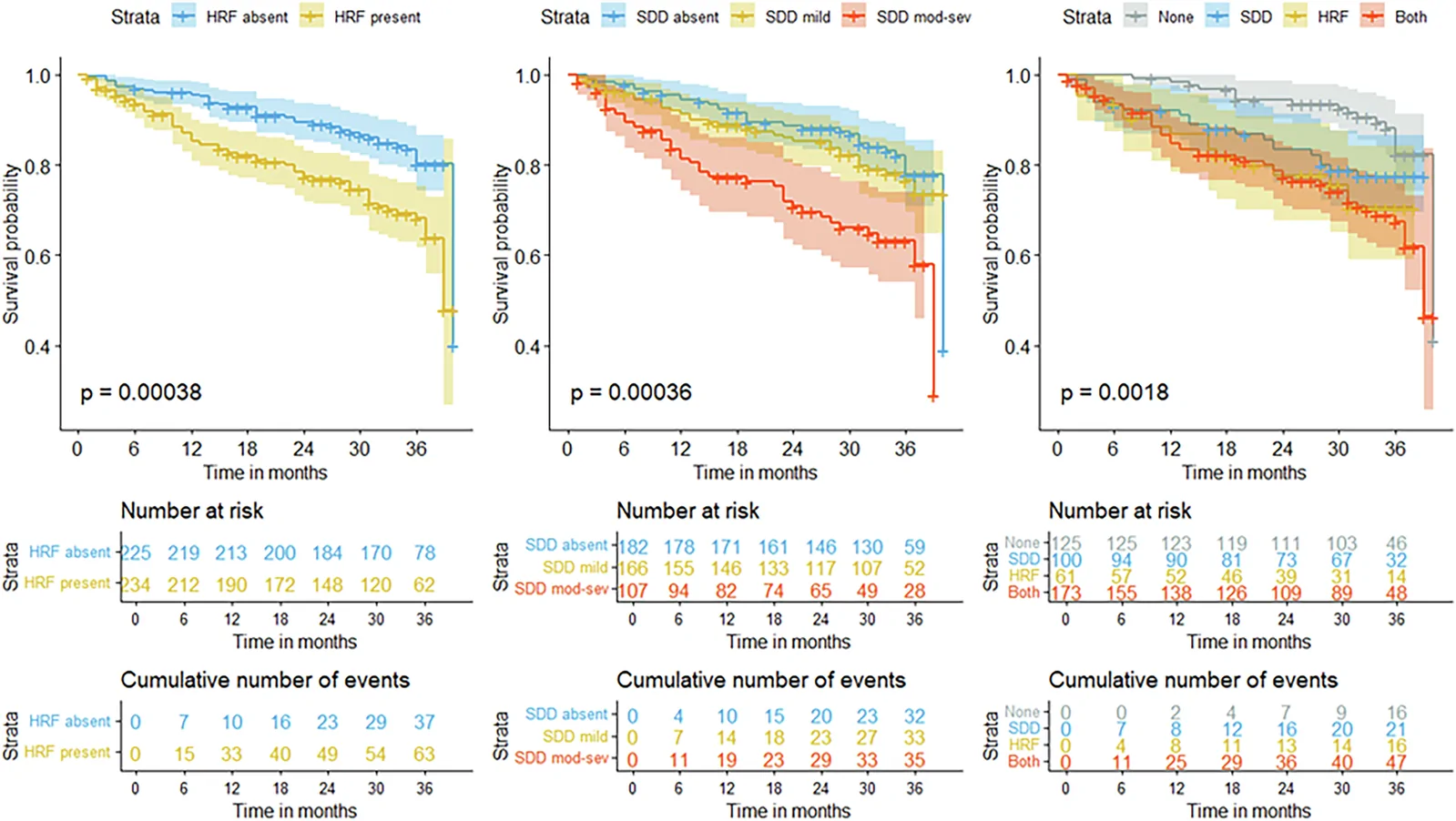
KM survival plots for HRF and SDD and Both. Kaplan Meier survival plots comparing time to development of eMNV by HRF **A** and SDD load **B**, mutually exclusive categories and combined **C**. HRF hyperreflective foci, SDD subretinal drusenoid deposit; mod-sev, moderate to severe.

The survival analysis for onset of any eMNV as outcome category and with time estimates at 90% and 50% survival probability, showed that presence of baseline HRF was associated with a noticeably shorter survival time (10 months, Interquartile Range 7.0, 13.0) compared to eyes without HRF (23 months, IQR 16.0, 32.0, *p* < 0.001, Fig. 2 and Table 8S). The presence of SDD also shortened survival times, with a moderate to severe load reducing survival to 6 months (IQR 4.0, 12) and a mild load to 15 months (IQR 10, 28) compared to eyes with no SDD which had the longest survival time of 19 months (IQR 15, 32; *p* < 0.001). For 50% survival probability significance was not reached, as the follow up was not long enough to extrapolate meaningful information.

**Fig. 3:**
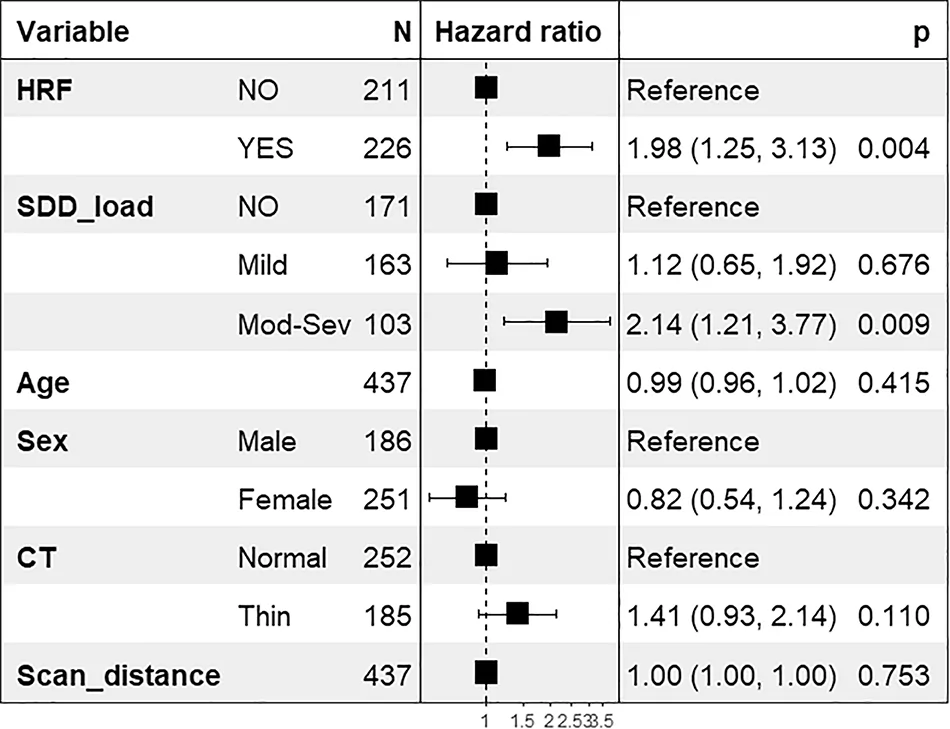
The hazard of progression to eMNV by HRF and SDD load. Multivariate Cox proportional hazard regression model assessing the hazard of progression to eMNV by HRF and SDD as predictor variables, adjusted for choroidal thickness and scan distance. Interaction term between predictors were tested and observed no significant interaction. HRF hyperreflective foci, SDD subretinal drusenoid deposit, Mod-Sev moderate to severe, CT choroidal thickness.

The forest plot of the multivariate Cox proportional hazard model (Fig. 3) shows a significantly increased hazard of progression to any eMNV in the presence of HRF (HR 1.98, range 1.25,3.13; *p* = 0.004) and a moderate to severe load of SDD (HR 2.14, range 1.21, 3.77; *p* = 0.009), while the point estimate for a mild SDD load was only marginally and non-significantly increased (HR 1.12, range 0.65, 1.92; *p* = 0.68). A thin choroid also showed only a marginally and non-significantly increased hazard (HR 1.41, range 0.93, 2.14; *p* = 0.11).

**Fig. 4:**
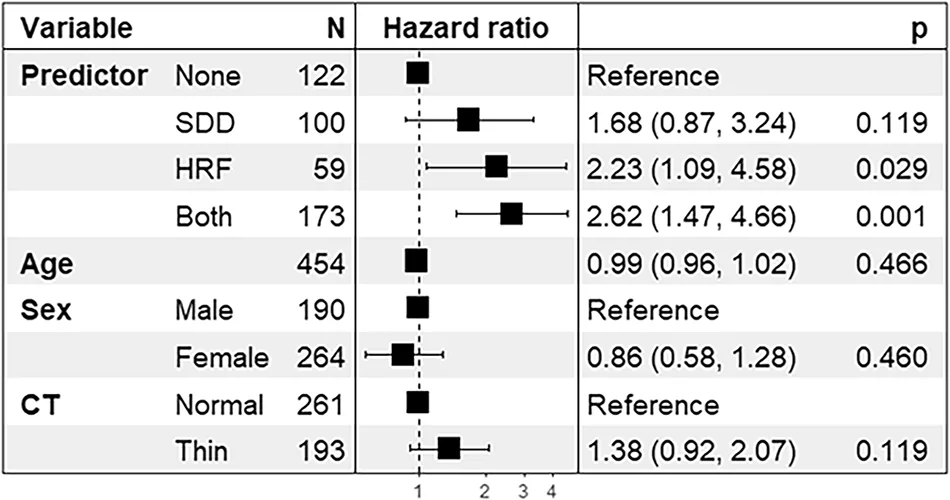
The hazard of progression to eMNV by mutually exclusive categories. Multivariate Cox proportional hazard regression model assessing the hazard of progression to eMNV by HRF and SDD as predictor variables. HRF hyperreflective foci, SDD subretinal druesenoid deposit, CT choroidal thickness.

Repeating the multivariate Cox proportional hazard model including mutually exclusive combined categories of HRF and SDD, and both (Fig. 4), we found the hazard of progression to any eMNV to be highest in eyes that demonstrated the combined presence of HRF and SDD (HR 2.62, range 1.47, 4.66; *p* = 0.001).

The cumulative incidence of eMNV was consistently higher in eyes with HRF at 12, 24, and 36 months compared to eyes without HRF, indicating earlier and more frequent progression. The cumulative incidence of eMNV also increased in a stepwise manner with greater SDD load, with the highest progression rates observed in eyes with moderate-to-severe SDD (Table 9S).

## Discussion

We performed an in-depth analysis on the association between HRF and SDD, and development of features of atrophy and exudation, based on longitudinal analysis of data from iAMD fellow eyes of patients with eMNV in the first eye. We subdivided our cohort into 4 distinct follow up groups; we then explored the associations of HRF and SDD with these 4 groups and made a number of notable observations. A moderate to severe SDD load doubled the hazard of progression to any eMNV compared to eyes without SDD. A significant gradient in rapidity of progression to eMNV was seen compared to eyes with no SDD, with increasingly faster rates of progression in higher SDD loads. A faster rate of progression to eMNV was also observed in eyes with HRF.

We observed that both close proximity and remoteness of HRF to regions of irregularly elevated RPE was associated with a higher frequency of FoA. In addition, we observed that most HRF were located in the outer retina and only a few eyes had HRF in inner retina only. This is not surprising as migrating RPE are more likely to be seen in the outer retina. Our data are supportive of previous observations that HRF presence and spatial distribution are factors in the development of atrophic foci arising from loss of outer retinal layers [20, 21].

We observed that baseline HRF are associated with marginally reduced BCVA over time and we provided an estimate of the change. Although the fall in BCVA was small, it is consistent with previous reports of reduced function in eyes exhibiting this feature and suggest that HRF might be associated with photoreceptor disruption [22]. Liu et al. showed an association between presence of HRF and cone mediated visual disfunction [23]. Lad et al. demonstrated that eyes with HRF develop deterioration in visual function over a two year period, which was detectable on cone contrast tests and microperimetry, although they did not observe detrimental effects on BCVA [24]. It is possible that differences in sample size and constitution between our study and that of Lad et al, as well as the longer follow up in our patient population (up to 3 years versus 2 years in the study by Lad et al.) may have allowed us to notice this marginal worsening in BCVA.

In addition, we examined the associations between SDD presence, spatial distribution and load on progression to exudation. To the best of our knowledge, the differential effects of SDD load on progression to eMNV has not yet been examined in high risk iAMD eyes. In our study the presence of few SDD (mild load) resulted in a marginally faster progression to eMNV compared to eyes without, whereas a moderate to severe load resulted in a significantly faster progression, hinting at the potential use of SDD as a biomarker in early AMD cases. Furthermore, our findings suggest that both HRF presence and higher SDD burden are associated with earlier disease progression.

Because we observed that both HRF and SDD were more likely to occur in the same eye, we undertook a Cox regression analysis with eMNV as the outcome variable and both HRF and SDD as the predictors. This model showed that the hazard ratio for HRF was highly statistically significant while SDD remained in the model with significance retained for a moderate to severe load only. When reassessing the model using mutually exclusive and combined categories of HRF and SDD, we found the hazard of progression to any eMNV to be highest in eyes that demonstrated the presence of both HRF and SDD. Larger sample sizes, quantitative estimates of the volumes of HRF and SDD through AI detection, along with a more prolonged follow up time may help define potential divergence in the pathways of progression to atrophy versus neovascularisation.

Our findings on the roles of HRF and SDD highlight a high degree of co-linearity of these two biomarkers. Nonetheless, regression analyses that included both of these biomarkers indicate that RPE loss, which at a clinical level reflects development of regions of focal atrophy and which is represented by HRF in the present study, is a critical step in the pathogenesis of eMNV. SDD, on the other hand, seem to have a stronger pathogenetic role in the development of atrophy without concomitant eMNV. It is therefore possible that SDD could be a biomarker for choroidal insufficiency and reflect a reduced ability of the choroidal vasculature for reparative angiogenesis. These findings also present opportunities to expand the present AMD staging systems in which the iAMD stage is currently defined (drusen ≥ 125 μm), encompassing a group of eyes with very low to very high risk of progression [25–28]. Notably, because iAMD eyes show vastly different rates of progression to eMNV and geographic atrophy, at which point there is already considerable vision loss, many eyes that reach such levels of AMD severity will not have the potential to recover already lost vision [7, 29–33]. It would be possible to use existing longitudinal datasets acquired within trials or from imaging databases constructed during routine care from patients with bilateral or unilateral iAMD to explore expansion of the iAMD stage, including whether there is presence of HRF and SDD as an initial step. However, given the development of AI algorithms which can segment and provide counts of HRF and volumes of SDD, as well as that of sub RPE (classical) drusen load, it is timely to also consider whether quantitative metrics can be incorporated within a highly granular iAMD staging system, and also consider a strategy to validate such a system.

Our study has several limitations. First, we did not grade for HRF load and with SDD, we merely had two categories of severity (mild and moderate to severe). Artificial intelligence algorithms were not available for this analysis and we therefore did not obtain absolute counts of HRF or numeric estimates of SDD. Second, we did not possess time to onset of FoA as a variable, since grading was only performed at baseline, point of conversion to eMNV and at the final follow-up visit and we could therefore not model this outcome. The EDNA study design was predicated on accurate detection of progression to exudative neovascular AMD. Images at conversion to exudation and at 18 and 36 months, which were the key additional assessment points, were submitted to the reading centre and form the repository. Grading for new onset of FoA at visits other than those captured at the repository was, therefore, not possible, which is why time to FoA development is not available. Furthermore, non-informative censoring of FoA is an untestable assumption in our dataset, and shared pathophysiological pathways between FoA and the OCT biomarkers examined (HRF, SDD) may introduce bias in the estimated hazard ratios. Prospective studies with timestamped ascertainment of all outcome transitions would therefore be needed. Third, the characteristics of the study sample (presence of nAMD in one eye with observations made on the fellow eye with intermediate AMD) preclude extrapolation of the findings to patients with bilateral intermediate AMD. Fifth, we did not have data on the whether atrophy was involving the fovea or not, therefore lacking precise information with respect to the potential adverse functional effects of FoA on BCVA.

Strengths of this study include accurate knowledge in terms of time to onset of eMNV and use of outputs from reading centre grading on HRF and SDD.

## Conclusion

Our study shows that HRF and SDD are important biomarkers that influence iAMD worsening, particularly in relation to atrophic changes. These findings highlight the potential value of incorporating HRF and SDD into a more granular iAMD staging system to improve risk stratification.

## Summary

### What was known before

- HRF and SDD are established OCT biomarkers linked to increased risk of progression from intermediate AMD to either geographic atrophy or neovascular AMD.
- The strength of these associations varies, and it has been unclear which features of HRF and SDD best predict whether high-risk eyes progress toward atrophy or exudation.

### What this study adds

- Identifies that both HRF and especially higher SDD load significantly accelerate progression to exudative MNV in high-risk fellow eyes, with moderate SDD load doubling the hazard.
- Suggests that some HRF characteristics, such as location, might be linked with a higher affinity to atrophy.
- Shows that HRF are associated with a measurable decline in visual acuity and that their spatial characteristics relate to atrophy development.
- Demonstrates that combining HRF and SDD characteristics improves risk stratification, supporting their inclusion in a more granular iAMD staging framework.

## Supplementary information

Table 1S

Table 2S

Table 3S

Table 4S

Table 5S

Table 6S

Table 7S

Table 8S

Table 9S

## Data Availability

The data that support the findings of this study are available from the corresponding author upon reasonable request.
