# Supplementary material for: Hyperreflective foci and subretinal drusenoid deposits as predictors of exudation in intermediate AMD eyes with contralateral neovascular AMD
Source: Eye (Lond). 2026 Jul 18;40(15):2360–6. doi: 10.1038/s41433-026-04617-8 (PMC13675177; doi:10.1038/s41433-026-04617-8)
Supplement: Supplementary file 1 [file 41433_2026_4617_MOESM1_ESM.docx]

**Table 1S** Frequency of SDD, load and distribution by outcome categories

| **OCT characteristics** | **Heidelberg**  N = 331^1^ | **Nidek**  N = 15^1^ | **Topcon**  N = 70^1^ | **Zeiss**  N = 43^1^ |
| --- | --- | --- | --- | --- |
| **Scan dimension** |  |  |  |  |
| 20 | 0 (0%) | 15 (100%) | 0 (0%) | 0 (0%) |
| 20x10 | 7 (2.1%) | 0 (0%) | 0 (0%) | 0 (0%) |
| 20x15 | 90 (27%) | 0 (0%) | 0 (0%) | 0 (0%) |
| 20x20 | 70 (21%) | 0 (0%) | 69 (99%) | 43 (100%) |
| 20x25 | 1 (0.3%) | 0 (0%) | 0 (0%) | 0 (0%) |
| 20x6 | 0 (0%) | 0 (0%) | 1 (1.4%) | 0 (0%) |
| 30x10 | 1 (0.3%) | 0 (0%) | 0 (0%) | 0 (0%) |
| 30x15 | 14 (4.2%) | 0 (0%) | 0 (0%) | 0 (0%) |
| 30x20 | 17 (5.1%) | 0 (0%) | 0 (0%) | 0 (0%) |
| 30x25 | 130 (39%) | 0 (0%) | 0 (0%) | 0 (0%) |
| 30x5 | 1 (0.3%) | 0 (0%) | 0 (0%) | 0 (0%) |
| **Scan density** |  |  |  |  |
| 7 | 1 (0.3%) | 0 (0%) | 1 (1.4%) | 0 (0%) |
| 12 | 0 (0%) | 15 (100%) | 0 (0%) | 0 (0%) |
| 13 | 7 (2.1%) | 0 (0%) | 0 (0%) | 0 (0%) |
| 19 | 82 (25%) | 0 (0%) | 0 (0%) | 0 (0%) |
| 25 | 75 (23%) | 0 (0%) | 0 (0%) | 0 (0%) |
| 31 | 120 (36%) | 0 (0%) | 0 (0%) | 0 (0%) |
| 37 | 23 (6.9%) | 0 (0%) | 0 (0%) | 0 (0%) |
| 49 | 12 (3.6%) | 0 (0%) | 0 (0%) | 0 (0%) |
| 61 | 9 (2.7%) | 0 (0%) | 0 (0%) | 0 (0%) |
| 128 | 2 (0.6%) | 0 (0%) | 69 (99%) | 43 (100%) |
