# Supplementary material for: Hyperreflective foci and subretinal drusenoid deposits as predictors of exudation in intermediate AMD eyes with contralateral neovascular AMD
Source: Eye (Lond). 2026 Jul 18;40(15):2360–6. doi: 10.1038/s41433-026-04617-8 (PMC13675177; doi:10.1038/s41433-026-04617-8)
Supplement: Supplementary file 2 [file 41433_2026_4617_MOESM2_ESM.docx]

Table 2S	Schoenfeld Residual Test for Proportional Hazards Assumption

| **Covariate** | **Chi-square** | **df** | **P value^a^** |
| --- | --- | --- | --- |
| HRF | 1.188 | 1 | 0.276 |
| SDD Load | 2.799 | 2 | 0.247 |
| Age | 0.003 | 1 | 0.958 |
| Sex | 8.259 | 1 | 0.004 |
| Choroidal Thickness | 0.481 | 1 | 0.488 |
| **GLOBAL** | **13.828** | **6** | **0.032** |
| ^a^P < 0.05 indicates a potential violation of the proportional hazards assumption. Sex showed a significant result (p = 0.004); see text for time-varying coefficient analysis. | | | |
