# Supplementary material for: Hyperreflective foci and subretinal drusenoid deposits as predictors of exudation in intermediate AMD eyes with contralateral neovascular AMD
Source: Eye (Lond). 2026 Jul 18;40(15):2360–6. doi: 10.1038/s41433-026-04617-8 (PMC13675177; doi:10.1038/s41433-026-04617-8)
Supplement: Supplementary file 3 [file 41433_2026_4617_MOESM3_ESM.docx]

Table 3S	Time-Varying Coefficient Models: Interaction with log(t)

| **Covariate** | **HR (exp coef)** | **SE** | **z** | **P value^a^** |
| --- | --- | --- | --- | --- |
| HRF × log(t) | 1.162 | 0.080 | 1.873 | 0.061 |
| SDD Load Mild × log(t) | 0.982 | 0.095 | -0.191 | 0.849 |
| SDD Load Moderate × log(t) | 1.194 | 0.100 | 1.770 | 0.077 |
| Sex × log(t) | 1.025 | 0.077 | 0.325 | 0.746 |
| ^a^A significant p-value indicates the hazard ratio for that covariate changes over time, confirming violation of the proportional hazards assumption. HR = hazard ratio per unit increase in the log(t) interaction term. | | | | |
