# Supplementary material for: Hyperreflective foci and subretinal drusenoid deposits as predictors of exudation in intermediate AMD eyes with contralateral neovascular AMD
Source: Eye (Lond). 2026 Jul 18;40(15):2360–6. doi: 10.1038/s41433-026-04617-8 (PMC13675177; doi:10.1038/s41433-026-04617-8)
Supplement: Supplementary file 4 [file 41433_2026_4617_MOESM4_ESM.docx]

**Table 4S** BCVA at Baseline and final visit

| **Outcome categories** | **N** | **iAMD without FoA**  N = 272 | **persistent or new onset FoA**  N = 83 | **eMNV**  N = 66 | **eMNV_FoA**  N = 38 | **p-value**^1^ |
| --- | --- | --- | --- | --- | --- | --- |
| **BCVA- Baseline** | 419 |  |  |  |  | <0.001 |
| Mean (SD) |  | 79.7 (5.4) | 77.8 (5.4) | 79.6 (5.2) | 76.6 (4.7) |  |
| Median (Q1, Q3) |  | 80.0 (75.0, 84.0) | 79.0 (73.0, 82.0) | 80.0 (75.0, 84.0) | 76.5 (73.5, 80.0) |  |
| Min, Max |  | 59.0, 94.0 | 68.0, 91.0 | 70.0, 89.0 | 68.0, 85.0 |  |
| **BCVA- final visit** | 419 |  |  |  |  | <0.001 |
| Mean (SD) |  | 79 (7) | 74 (10) | 76 (9) | 74 (8) |  |
| Median (Q1, Q3) |  | 80 (76, 84) | 77 (69, 82) | 78 (72, 83) | 76 (69, 80) |  |
| Min, Max |  | 43, 95 | 40, 90 | 42, 96 | 55, 85 |  |

The table shows the mean (standard deviation), median (inter quartile range) and minimum and maximum value of BCVA across final categories. Abbreviation: BCVA, best corrected visual acuity; iAMD, intermediate age-related macular degeneration; FoA, focal atrophy; eMNV, exudative macular neovascularization; HRF, hyper reflective material;

^1^Kruskal-Wallis rank sum test
