# Supplementary material for: Hyperreflective foci and subretinal drusenoid deposits as predictors of exudation in intermediate AMD eyes with contralateral neovascular AMD
Source: Eye (Lond). 2026 Jul 18;40(15):2360–6. doi: 10.1038/s41433-026-04617-8 (PMC13675177; doi:10.1038/s41433-026-04617-8)
Supplement: Supplementary file 5 [file 41433_2026_4617_MOESM5_ESM.docx]

**Table 5S** Progression to eMNV by HRF features

|  |  | **All** | **By progression to any eMNV** | | |
| --- | --- | --- | --- | --- | --- |
|  | **N** | **N = 459**^1^ | **N** | **NO**  N = 355^1^ | **YES**  N = 104^1^ |
| **HRF** | 459 |  | 459 |  |  |
| NO |  | 225 (49.0%) |  | 187 (52.7%) | 38 (36.5%) |
| YES |  | 234 (51.0%) |  | 168 (47.3%) | 66 (63.5%) |
| **HRF_IE_relation** | 171 |  | 171 |  |  |
| Adjacent |  | 67 (39.2%) |  | 51 (43.2%) | 16 (30.2%) |
| Remote |  | 37 (21.6%) |  | 24 (20.3%) | 13 (24.5%) |
| Both |  | 67 (39.2%) |  | 43 (36.4%) | 24 (45.3%) |
| **HRF_location** | 234 |  | 234 |  |  |
| Inner retina |  | 5 (2.1%) |  | 3 (1.8%) | 2 (3.0%) |
| Outer retina |  | 157 (67.1%) |  | 113 (67.3%) | 44 (66.7%) |
| Both |  | 72 (30.8%) |  | 52 (31.0%) | 20 (30.3%) |

The table show the prevalence and characteristics of HRF for the total cohort and by progression to eMNV. Abbreviation: eMNV, exudative macular neovascularization; HRF, hyperreflective foci; IE, irregular elevation of RPE;

^1^n (%); ^2^Pearson's Chi-squared test, Fisher's exact test
