# Supplementary material for: Hyperreflective foci and subretinal drusenoid deposits as predictors of exudation in intermediate AMD eyes with contralateral neovascular AMD
Source: Eye (Lond). 2026 Jul 18;40(15):2360–6. doi: 10.1038/s41433-026-04617-8 (PMC13675177; doi:10.1038/s41433-026-04617-8)
Supplement: Supplementary file 6 [file 41433_2026_4617_MOESM6_ESM.docx]

**Table 6S** Frequency of SDD and its OCT characteristics by progression to any eMNV

|  | **All** | | **By progression to eMNV** | | |
| --- | --- | --- | --- | --- | --- |
|  | **N** | **N = 459**^1^ | **N** | **NO**  N = 355^1^ | **YES**  N = 104^1^ |
| **SDD** | 455 |  | 455 |  |  |
| NO |  | 182 (40.0%) |  | 149 (42.5%) | 33 (31.7%) |
| YES |  | 273 (60.0%) |  | 202 (57.5%) | 71 (68.3%) |
| **SDD load** | 273 |  | 273 |  |  |
| Mild |  | 166 (60.8%) |  | 132 (65.3%) | 34 (47.9%) |
| Moderate |  | 107 (39.2%) |  | 70 (34.7%) | 37 (52.1%) |
| **SDD_proximity to IE** | 169 |  | 169 |  |  |
| Remote |  | 117 (69.2%) |  | 85 (71.4%) | 32 (64.0%) |
| Both |  | 52 (30.8%) |  | 34 (28.6%) | 18 (36.0%) |
| **SDD_location** | 272 |  | 272 |  |  |
| Extra-foveal |  | 225 (82.7%) |  | 169 (84.1%) | 56 (78.9%) |
| Both |  | 47 (17.3%) |  | 32 (15.9%) | 15 (21.1%) |
| ^1^n (%) | | | | | |

The table shows OCT characteristics of SDD, such as SDD load, its relation to IE and SDD location. Abbreviation: iAMD, intermediate age-related macular degeneration; FoA, focal atrophy; eMNV, exudative macular neovascularization; SDD, subretinal drusenoid deposit; ^1^n (%), ^2^Pearson's Chi-squared test; Fisher's exact test

Abbreviation: eMNV, exudative macular neovascularisation; SDD, subretinal drusenoid deposit; IE, irregular elevation of the retinal pigment epithelium; ^1^n (%);
