# Supplementary material for: Hyperreflective foci and subretinal drusenoid deposits as predictors of exudation in intermediate AMD eyes with contralateral neovascular AMD
Source: Eye (Lond). 2026 Jul 18;40(15):2360–6. doi: 10.1038/s41433-026-04617-8 (PMC13675177; doi:10.1038/s41433-026-04617-8)
Supplement: Supplementary file 7 [file 41433_2026_4617_MOESM7_ESM.docx]

**Table 7S** Frequency of SDD, load and distribution by outcome categories

| **Outcome categories** | **N** | **iAMD without FoA** N = 272^1^ | **persistent or new onset FoA**  N = 83^1^ | **eMNV**  N = 66^1^ | **eMNV_FoA**  N = 38^1^ |
| --- | --- | --- | --- | --- | --- |
| **SDD** | 455 |  |  |  |  |
| NO |  | 132 (49.1%) | 17 (20.7%) | 26 (39.4%) | 7 (18.4%) |
| YES |  | 137 (50.9%) | 65 (79.3%) | 40 (60.6%) | 31 (81.6%) |
| **SDD load** | 273 |  |  |  |  |
| Mild |  | 97 (70.8%) | 35 (53.8%) | 22 (55.0%) | 12 (38.7%) |
| Moderate to Severe |  | 40 (29.2%) | 30 (46.2%) | 18 (45.0%) | 19 (61.3%) |
| **SDD_IE_relation** | 169 |  |  |  |  |
| Remote |  | 56 (83.6%) | 29 (55.8%) | 17 (60.7%) | 15 (68.2%) |
| Both |  | 11 (16.4%) | 23 (44.2%) | 11 (39.3%) | 7 (31.8%) |
| **SDD_location** | 273 |  |  |  |  |
| Sub-foveal |  | 1 (0.7%) | 0 (0.0%) | 0 (0.0%) | 0 (0.0%) |
| Extra-foveal |  | 117 (85.4%) | 52 (80.0%) | 31 (77.5%) | 25 (80.6%) |
| Both |  | 19 (13.9%) | 13 (20.0%) | 9 (22.5%) | 6 (19.4%) |

Abbreviation: iAMD, intermediate age-related macular degeneration; FoA, focal atrophy; eMNV, exudative macular neovascularization; SDD, subretinal drusenoid deposit; IE, irregular elevation; ^1^n (%);
