# Supplementary material for: Hyperreflective foci and subretinal drusenoid deposits as predictors of exudation in intermediate AMD eyes with contralateral neovascular AMD
Source: Eye (Lond). 2026 Jul 18;40(15):2360–6. doi: 10.1038/s41433-026-04617-8 (PMC13675177; doi:10.1038/s41433-026-04617-8)
Supplement: Supplementary file 8 [file 41433_2026_4617_MOESM8_ESM.docx]

**Table 8S** Age- and Sex-Adjusted Survival Times at 50% and 90% Survival Probability

| **Variable** | **Level** | **50% Survival Time** | **90% Survival Time** | **P value (adjusted)^a^** |
| --- | --- | --- | --- | --- |
|  |  | **months (95% CI)** | **months (95% CI)** |  |
| **HRF** | NO | Not reached | 19 (13–33) | <0.001 |
|  | YES | 40 (NA–39) | 10 (6–14) |  |
| **SDD load** | NO | 40 (NA–40) | 19 (12–31) | <0.001 |
|  | Mild | 40 (NA–39) | 13 (10–25) |  |
|  | Moderate to Severe | 39 (NA–31) | 7 (4–12) |  |
| ^a^Wald p-value (HRF) and likelihood ratio test p-value (SDD Load) from separate Cox models adjusted for Age and Sex. Survival times estimated at mean Age and modal Sex (Male). Abbreviations: HRF, hyperreflective foci; SDD, subretinal drusenoid deposit | | | | |
