# Supplementary material for: Hyperreflective foci and subretinal drusenoid deposits as predictors of exudation in intermediate AMD eyes with contralateral neovascular AMD
Source: Eye (Lond). 2026 Jul 18;40(15):2360–6. doi: 10.1038/s41433-026-04617-8 (PMC13675177; doi:10.1038/s41433-026-04617-8)
Supplement: Supplementary file 9 [file 41433_2026_4617_MOESM9_ESM.docx]

**Table 9S Cumulative Incidence of eMNV by SDD and HRF**

| Grouping | Stratum | Time (months) | N at risk | Cumulative events | Cumulative Incidence (95% CI) |
| --- | --- | --- | --- | --- | --- |
| **HRF** | *HRF absent* | 12 | 213 | 10 | 4.5% (1.7%–7.1%) |
|  |  | 24 | 184 | 23 | 10.5% (6.4%–14.5%) |
|  |  | 36 | 78 | 37 | 19.8% (13.4%–25.6%) |
|  | *HRF present* | 12 | 190 | 33 | 14.7% (10.0%–19.3%) |
|  |  | 24 | 148 | 49 | 22.6% (16.8%–28.0%) |
|  |  | 36 | 62 | 63 | 31.9% (24.7%–38.4%) |
| **SDD Load** | *SDD absent* | 12 | 171 | 10 | 5.5% (2.1%–8.8%) |
|  |  | 24 | 146 | 20 | 11.3% (6.5%–15.8%) |
|  |  | 36 | 59 | 32 | 22.1% (14.4%–29.1%) |
|  | *SDD mild* | 12 | 146 | 14 | 8.7% (4.2%–12.9%) |
|  |  | 24 | 117 | 23 | 14.8% (9.0%–20.2%) |
|  |  | 36 | 52 | 33 | 23.4% (15.8%–30.4%) |
|  | *SDD Mod-Sev* | 12 | 82 | 19 | 18.5% (10.6%–25.7%) |
|  |  | 24 | 65 | 29 | 29.2% (19.6%–37.7%) |
|  |  | 36 | 28 | 35 | 36.8% (26.1%–45.9%) |
| **HRF & SDD Combined** | *Neither* | 12 | 123 | 2 | 1.6% (0.0%–3.8%) |
|  |  | 24 | 111 | 7 | 5.7% (1.5%–9.7%) |
|  |  | 36 | 46 | 16 | 17.7% (8.6%–25.8%) |
|  | *SDD only* | 12 | 90 | 8 | 8.0% (2.5%–13.2%) |
|  |  | 24 | 73 | 16 | 16.7% (8.8%–23.8%) |
|  |  | 36 | 32 | 21 | 22.5% (13.5%–30.6%) |
|  | *HRF only* | 12 | 52 | 8 | 13.3% (4.3%–21.5%) |
|  |  | 24 | 39 | 13 | 22.2% (10.8%–32.3%) |
|  |  | 36 | 14 | 16 | 29.5% (15.8%–40.9%) |
|  | *Both HRF & SDD* | 12 | 138 | 25 | 15.3% (9.6%–20.6%) |
|  |  | 24 | 109 | 36 | 22.8% (15.9%–29.1%) |
|  |  | 36 | 48 | 47 | 32.6% (24.1%–40.1%) |

Cumulative incidence of eMNV at 12, 24, and 36 months by HRF status, SDD load, and combined HRF/SDD category

Abbreviations: HRF, hyperreflective foci; SDD, subretinal drusenoid deposit; mod-sev, moderate to severe
